# Concizumab prophylaxis in a 2-year-old patient with severe hemophilia B and inhibitor: a case report

**DOI:** 10.3389/fphar.2026.1876168

**Published:** 2026-07-15

**Authors:** Mandy Yuan, Sanober Nusrat, Osman Khan

**Affiliations:** 1 The University of Oklahoma College of Medicine, Oklahoma City, OK, United States; 2 Division of Hematology-Oncology, Department of Internal Medicine, The University of Oklahoma Health Campus, Oklahoma City, OK, United States; 3 Division of Pediatric Hematology-Oncology, Department of Pediatrics, The University of Oklahoma Health Campus, Oklahoma City, OK, United States

**Keywords:** concizumab, factor IX inhibitors, hemophilia B, non-factor therapy, tissue factor pathway inhibitor

## Abstract

Hemophilia B is a rare X-linked congenital bleeding disorder characterized by a deficiency in coagulation factor IX (FIX). Standard management relies on exogenous factor replacement; however, the development of neutralizing alloantibodies (inhibitors) against infused clotting factor can significantly reduce therapeutic efficacy and complicate long-term management. Consequently, patients turn to bypassing agents or non-factor therapies to help achieve adequate hemostatic control. Concizumab is a novel subcutaneous non-factor therapy for hemophilia A and B that targets the tissue factor pathway inhibitor (TFPI). While it is approved for patients >12 years old, data remains sparse in younger children. Prophylactic treatment with concizumab reduces several limitations associated with inhibitor development and variability in treatment response with conventional therapies, especially in pediatric populations. We report a toddler with hemophilia B who developed a low-titer inhibitor following treatment with recombinant coagulation factor IX agents, resulting in recurrent bleeding complications and consistent subtherapeutic hemostatic control on standard bypassing agents. The patient was transitioned to concizumab for long-term prophylaxis, with dose adjustments based on clinical response and concizumab drug levels. Following the initiation of concizumab, the patient demonstrated a marked reduction in bleeding episodes. We highlight an individualized early drug-level-guided dose escalation approach in a very young child, demonstrating that pharmacokinetic monitoring can be utilized proactively to optimize therapeutic response in this age group, leading to effective drug levels and excellent clinical response. This case supports the role of concizumab in potentially reducing treatment burden and improving hemostatic outcomes in young patients with severe hemophilia B and inhibitors.

## 1 Introduction

Hemophilia B, also known as Factor IX (FIX) deficiency, is a rare X-linked congenital bleeding disorder caused by insufficient clotting factor IX. Disease severity ranges from mild to severe, with severe deficiency defined as having less than 1% of residual clotting factor activity (Chowdary et al., 2025). FIX is a vitamin K-dependent protease that works in the intrinsic branch of the coagulation pathway, and mutations across the F9 gene including early distinct functional domains, such as the γ-carboxyglutamic acid (Gla) domain, can cause hemophilia B through multiple mechanisms (Larson et al., 1996; Ivanciu and Camire, 2025).

Traditional therapies for hemophilia B rely on factor replacement, temporarily restoring the body’s normal clotting function. Although this remains the gold standard for prophylactic and on-demand treatment, it does not come without its challenges (Castaman et al., 2025). These factor-based therapies are limited by their relatively short half-lives, frequent intravenous administrations, and the consequent need for central venous access devices (CVADs), such as peripherally inserted central catheters or ports, in young patients. These devices expose children to higher risks of infections, sepsis, and thrombotic events (Peyvandi et al., 2013). Adherence and family support for these consistent infusions can pose challenges to maintaining prophylactic regimens (Miesbach et al., 2024).

Another major limitation is the development of neutralizing antibodies–termed inhibitors–against infused clotting factors, which occurs in approximately 3%–10% of patients with hemophilia B (Ljung et al., 2019; Alexander et al., 2022; Swan et al., 2022). Although less common than in hemophilia A, inhibitor development in hemophilia B is associated with more severe complications including anaphylaxis and nephrotic syndrome during exposure to FIX products (Santoro et al., 2018; Chowdary et al., 2025), further limiting treatment options. Inhibitors are described as neutralizing high-affinity antibodies, primarily IgG, directed against exogenous clotting factor therapies (Ljung et al., 2019; Tieu et al., 2020; Swan et al., 2022). They are classified by their amount as low- (<5 Bethesda Units [BU]/mL) or high- (≥5 BU) titer. This renders factor replacement therapies ineffective; consequently, patients often require bypassing agents, such as recombinant factor VIIa or activated prothrombin complex concentrate, in addition to non-factor therapies.

Non-factor therapies have emerged to restore hemostatic balance without relying on exogenous FIX. Concizumab, a humanized monoclonal immunoglobulin G4 antibody, is a novel subcutaneous prophylactic agent for hemophilia A and B that targets the Kunitz-2 domain of tissue factor pathway inhibitor (TFPI), blocking its inhibitory activity and thereby increasing thrombin production through the extrinsic pathway (Shapiro, 2021; Castaman et al., 2025). In FIX deficiency, where the intrinsic pathway is compromised, this mechanism becomes particularly pertinent. Within the Explorer Program, multiple clinical trials evaluated the effectiveness and safety of concizumab, demonstrating significant efficacy in inhibitor populations with an approximately 86% decrease in mean annualized bleeding rate (ABR) compared to the control group [ABR 1.7 (95% CI: 1.0–2.9) vs. 11.8 (95% CI: 7.0–19.9)] (Castaman et al., 2025). Three non-fatal thromboembolic events prompted a protocol revision to lower daily dosing, after which no further events occurred (Castaman et al., 2025). Critically, concizumab is administered prophylactically and subcutaneously, reducing venous access burden, meaningful characteristics to reducing treatment burden in young children.

This report discusses a young pediatric patient with severe hemophilia B whose course was complicated by inhibitor development. Various treatment challenges led to the transition to prophylactic concizumab therapy, emphasizing its potential to mitigate the burdens in high-risk hemophilic patients.

## 2 Case presentation

We report the case of a 27-month-old male patient with severe hemophilia B (baseline FIX <1% activity) who is currently being managed at our Hemophilia Treatment Center at The University of Oklahoma. Previous genetic analysis revealed a nonsense mutation in the Gla region of the F9 gene, c.223C>T, resulting in a premature stop codon, p.Arg75Ter.

At 12 months of age, the patient was initiated on prophylactic treatment with extended half-life FIX, eftrenonacog alfa (Alprolix) dosed at 90 U/kg weekly, consistent with the FDA-approved dosing range for eftrenonacog alfa (50–100 IU/kg). This reflects the higher clearance rates and larger volume of distribution characteristically observed in pediatric patients with hemophilia B, as demonstrated in the Kids B-LONG clinical trial (Fischer et al., 2017). After developing a significant allergic reaction presenting as a skin rash and cough on exposure day 15, he was switched to nonacog beta pegol (Rebinyn) dosed at 178 U/kg. An inhibitor level was obtained 4 days prior to the reaction as part of routine monitoring but no immediate post-reaction measurement was performed. The higher dose of nonacog beta pegol was selected based on the patient’s relatively low peak FIX levels, rapid clearance on prior therapy, and an uncertain and evolving inhibitor status. Persistent suboptimal responses to the weekly intravenous infusions of nonacog beta pegol, characterized by the co-occurrence of unfavorable pharmacokinetic parameters, specifically low peak FIX levels following infusion and rapid clearance, and ongoing clinical bleeding episodes, prompted inhibitor testing on Exposure Day 4, which showed FIX inhibitor titer elevated at 3 BU. The patient was approximately 17 months old at this time.

With this new low-titer inhibitor diagnosis on Exposure Day 4 of nonacog beta pegol, the patient was transitioned to 272 U/kg of standard half-life FIX, nonacog gamma (Rixubis) up to 3 times weekly. Peripheral intravenous access was complicated by multiple hematomas and central access was deemed necessary at this time. Nonacog gamma infusions continued to be associated with poorly sustained FIX activity levels at 24–48 h. Furthermore, inhibitor levels peaked at 4.8 BU at 4 weeks of diagnosis.

A week after an infusaport was placed, the patient developed a catheter-associated methicillin-resistant *Staphylococcus aureus* (MRSA) bacteremia, requiring inpatient surgical debridement and aggressive hemostasis. During his inpatient stay, he developed a significant allergic reaction to nonacog gamma and was switched to recombinant coagulation factor VIIa (rFVIIa, eptacog alfa) for hemostasis. The rFVIIa (NovoSeven) dose was initially 90 mcg/kg, given every 3–8 h, which was escalated for management of bleeding complications. After medical optimization, the patient was subsequently discharged on continued prophylaxis of rFVIIa 166 mcg/kg once daily. While on rFVIIa, the patient continued to have severe bruising and developed a hematoma at the port site. This was managed with more frequent administration of rFVIIa and subsequent resolution, only for his clinical picture to be complicated with a posterior thigh hematoma about a week later.

In view of his suboptimal hemostatic response and continued development of soft tissue bleeding events, muscle hematomas, and port access challenges, the patient was started off-label on concizumab, 1 mg/kg on day 1 (loading dose), and then 0.2 mg/kg daily, for hemostatic management. The patient’s weight was 14.4 kg. The initiation of concizumab was undertaken following pharmacy approval with thorough clinical justification, given the patient’s complex clinical course including FIX allergy, inhibitor development, port infection, and failure of rFVIIa prophylaxis. The decision reflected a multidisciplinary clinical assessment weighing the known risks of off-label usage against the significant and ongoing bleeding burden in the patient. Informed consent for off-label use was obtained from the caregivers, who were counseled regarding the investigational nature of concizumab in children under 12 years of age, the available safety and efficacy data, and the absence of pediatric label approval.

Drug-level monitoring was performed using Concizumab Enzyme-Linked Immunosorbent Assay (ELISA), with samples collected immediately prior to the scheduled next dose (trough levels), beginning 4 weeks after treatment initiation. All samples were obtained in accordance with this guideline, and subsequent measurements were taken after 8 weeks on the same maintenance dose to confirm steady-state concentrations. Concizumab drug levels were subtherapeutic (<122.63 ng/mL) at dose of 0.2 mg/kg/day, which was subsequently increased to 0.25 mg/kg/day, with drug levels at the low-normal level (153.24 ng/mL, initial trough). This level fell below the label threshold of 200 ng/mL, thus the dose was escalated to 0.3 mg/kg/day and resulted in a therapeutic level (2909.9 ng/mL; target range 200–4000 ng/mL). Although this dosing exceeds current FDA-approved recommendations, the decision was made in the context of limited pediatric data, persistent pharmacokinetic variability, and the patient’s prior severe bleeding phenotype.

Following the transition to concizumab, over an observation period of approximately 7 months, the patient demonstrated a marked clinical improvement beginning after 3–4 weeks of treatment initiation, approximately at 21 months of age. The patient experienced zero breakthrough bleeding episodes, including complete resolution of muscle hematomas, bruising, and IV site bleeding. He has not required any rFVIIa (NovoSeven) since starting concizumab, in contrast to multiple doses required prior to the transition. Additionally, no transfusions have been needed following concizumab initiation. With the recent transition to concizumab and the need to ensure sustained clinical stability, central venous access via the port has been maintained in the near term. Discontinuation of the port will be considered as the patient continues to demonstrate clinical stability on concizumab therapy.

The patient underwent ongoing safety monitoring, specifically liver function tests were performed and remained within normal limits throughout the treatment course. The patient has remained clinically well throughout approximately 7 months of therapy with no signs and symptoms of thromboembolic events, and available laboratory results have not raised safety concerns. Anti-drug antibody testing was not pursued given the patient’s robust clinical response and zero breakthrough episodes, which did not warrant further investigation at this time. Should breakthrough bleeding occur, the planned rescue strategy is rFVIIa (NovoSeven) at a reduced dose due to the potentiating hemostatic effect of concizumab and the associated thrombotic risk with concurrent bypassing agent usage. Given the favorable clinical response and absence of adverse events, continuation at this dose was deemed appropriate with continued monitoring.

### 2.1 Family perspective

The family has reported a dramatic improvement in the patient’s quality of life since transitioning to concizumab and expressed being extremely happy with the overall response to the treatment. The reduction in caregiver anxiety, previously driven by frequent bleeding episodes, inhibitor concerns, port management, and rFVIIa failures, has been substantial.

## 3 Discussion

This case illustrates the extreme complexities of managing severe hemophilia B with inhibitors in a young toddler, particularly in the setting of difficult venous access and poor response to standard therapies. Inhibitor development immediately limits the effectiveness of conventional FIX replacement, even at elevated doses, due to the formation of high-affinity neutralizing IgG antibodies directed against the functional domains of the FIX protein. In this patient, low-titer inhibitor levels were confirmed following clinical reassessment after an allergic reaction to eftrenonacog alfa (Alprolix) on exposure day 15. Although an inhibitor level had been obtained as part of routine monitoring 4 days prior to the reaction, repeat testing was not performed immediately afterward, and the subsequent assessment occurred approximately 1 month later. This gap in post-reaction inhibitor evaluation represents a limitation, particularly given the well-recognized association between inhibitor development and allergic reactions to FIX products in early exposure days. Minimal response to recombinant FIX necessitated the use of bypassing agents such as rFVIIa. While rFVIIa does promote thrombin generation through the tissue factor-dependent activation of factor X, breakthrough bleeding from suboptimal hemostasis and allergic reactions further restricted available options that could reduce infusion burden.

These limitations highlight the crucial role of non-factor rebalancing therapies in the hemophilia with inhibitors population. Concizumab was selected after suboptimal responses and allergic reactions to alternate therapies. Because concizumab targets the Kunitz-2 domain of TFPI, preventing TFPI-mediated inhibition of FXa, its mechanism remains independent of circulating inhibitors and FIX. Although currently FDA-approved for patients of 12 years and older, concizumab remains one of the few therapies with proven efficacy in both hemophilia A and B with inhibitor populations (FDA, 2024). In contrast, alternative rebalancing agents such as fitusiran suppress antithrombin via RNA interference, altering thrombin regulation. Fitusiran has demonstrated robust bleeding rate reductions across inhibitor and non-inhibitor populations but has been reported to increase the risk of thrombosis, hepatotoxicity, and gallbladder complications, further underscoring the need for safer options in very young children (Srivastava et al., 2023; Young et al., 2023). Another anti-TFPI agent, marstacimab, approved in 2024, similarly lacks pediatric data under 12 years old. Importantly, no approved rebalancing agent has established safety or efficacy data in toddlers or young children, with pediatric trials still ongoing. Long-term safety in this age group remains uncertain. A recently published case series showcases a favorable safety profile and prophylactic effectiveness for concizumab in young children with severe hemophilia B and inhibitors (Levy-Mendelovich et al., 2026). This case report adds to the growing but still limited literature supporting concizumab usage in children younger than 12 years old.

In this young pediatric patient, early drug level monitoring and stepwise dose escalation revealed subtherapeutic levels at lower doses, with therapeutic levels achieved at a non-FDA recommended dose of 0.3 mg/kg/day. The off-label use of concizumab following multidisciplinary clinical assessment, informed consent from caregivers, and special pharmacy approval reflects a carefully considered real-world clinical decision-making process.

The subsequent disproportionate rise to 2909.9 ng/mL of concizumab drug level following a modest dose increase from 0.25 mg/kg/day to 0.3 mg/kg/day likely reflects non-linear pharmacokinetics in a low-bodyweight (14.4 kg), actively growing toddler. Per the label, apparent clearance and volume of distribution decreases with increased body weight, which may have contributed to drug accumulation as the patient gained weight over this period (Novo Nordisk, 2025). It is important to acknowledge that the 200–4,000 ng/mL target range for concizumab is derived from studies in patients ≥12 years, and its application in a toddler represents an extrapolation, limiting the interpretability of the study findings. This highlights a novel, patient-specific early drug-level-guided dosing strategy distinct from currently established approaches in older (≥12 years) populations. It showcases that pharmacokinetic monitoring of concizumab can be utilized proactively to optimize treatment response in children younger than 12 years old.

Marked clinical improvement and no adverse effects have been reported to date, with ongoing clinical and laboratory monitoring. Although liver functions remained within normal limits and no clinical evidence of thromboembolic events was observed during the treatment course and continued monitoring, formal protocol-driven laboratory monitoring per drug guidelines (including D-dimer) was not systematically obtained. This incomplete safety monitoring is acknowledged as a limitation of this report. Additionally, while a formalized ABR calculation is limited by the relatively short follow-up duration, the contrast between pre- and post-concizumab clinical course is clinically meaningful. Long-term follow-up data will be necessary to fully characterize the durability of the response.

Venous access complications in toddlers with hemophilia add another layer of complexity. Small veins, fragile tissue, and impaired ability to clot make maintaining peripheral intravenous access technically difficult and often traumatic. Central catheters tend to introduce additional risks of infection, thrombosis, and port malfunction, all of which occurred in our patient (Bedoya et al., 2020). Infections are known to have a higher incidence in hemophilic children with inhibitors (Ljung, 2004; Santagostino and Mancuso, 2010; Rodriguez et al., 2015) with a study of severe hemophilia A patients with inhibitors finding 41% of their participants developing CVAD infections, with Gram-positive infections occurring in significantly younger populations than Gram-negative infections (median 17 vs. 25 months respectively, P < 0.0001) (Rodriguez et al., 2015). These challenges underscore the need for therapies that bypass the reliance on venous access. Concizumab’s subcutaneous route of administration directly addresses these obstacles by eliminating frequent venous access, reducing procedure-related discomfort, and minimizing central line dependence. Patient preference data support this approach, with subcutaneous prophylaxis associated with higher mean utility scores (CI 95%) at 0.90 [0.87–0.93] compared to intravenous prophylaxis at 0.81 [0.78–0.85] (Johnston et al., 2021). Together with studies demonstrating more positive health-related quality of life effects of prophylaxis treatment over on-demand treatment (Usuba et al., 2019), this subcutaneous prophylactic approach is likely to meaningfully reduce caregiver anxiety and enhance overall wellbeing in this population.

Genetically, the patient’s nonsense mutation (c.223C>T; p.Arg75Ter) in the F9 gene truncates the protein within the Gla domain, disrupting Ca^2+^-dependent folding and preventing formation of downstream functional domains, which results in loss of FIX function and severe hemophilia B (Ivanciu and Camire, 2025). Truncating mutations in early domains have been associated with increased risk of inhibitor development (Liu et al., 2023). This genetic profile may therefore support early consideration of non-factor therapies such as concizumab.

Overall, this case demonstrates how concizumab can substantially reduce treatment burden in very young patients who are unable to tolerate frequent intravenous infusions, have difficulty maintaining central access, and develop inhibitors that render factor therapy ineffective. Utilizing an early drug-level-guided dosing strategy with concizumab has potential to minimize complications and improve quality of life for pediatric patients with complex hemophilia A and B.

## 4 Conclusion

This report highlights the significant complexities of managing severe hemophilia B with inhibitors in young toddlers. With key morbidities of CVADs, higher infection risk, and allergic reactions to numerous therapeutic agents, management of hemophilia B remains challenging. The development of non-factor therapies, such as concizumab, shows potential benefits for the management of hemophilia B in young patients; however, long-term outcomes in children younger than 12 years old remain uncertain. Future studies are warranted to evaluate the efficacy and safety of concizumab in this younger age group and explore patient-specific early drug-level-guided dosing strategies. Furthermore, the observed genetic profile generates the hypothesis that first-line use of non-factor therapies in patients with genetic predisposition to inhibitor development may decrease the likelihood of developing inhibitors, which calls for evaluation in prospective studies.

## 5 Summary

We report a pediatric patient with severe hemophilia B who developed inhibitors following standard factor treatments and subsequently transitioned to concizumab for long-term prophylaxis management. Despite initial subtherapeutic drug levels at standard dosages, favorable clinical response and a therapeutic drug level were achieved at a higher dose following early drug-level-guided dose assessment and escalation, demonstrating concizumab’s strong potential in managing severe hemophilia B with inhibitors in patients younger than 12 years old. Dissemination of this effective individualized dosing approach can further inform the limited data currently available on the efficacy of concizumab and pediatric hemophilia treatment options in inhibitor populations.

## Data Availability

The original contributions presented in the study are included in the article/Supplementary Material, further inquiries can be directed to the corresponding author.
